# Ultraslow Thrombolysis of Mechanical Tricuspid Prosthetic Thrombosis

**DOI:** 10.1016/j.jaccas.2025.104211

**Published:** 2025-07-16

**Authors:** Uzma Afzal, Jeffery Lau, Jonathan Yap, Jiang Ming Fam, Inn Jia Ooi, Kamalesh Anbalakan, Ju Le Tan, Zee Pin Ding, See Hooi Ewe, Kay Woon Ho

**Affiliations:** Department of Cardiology, National Heart Centre Singapore, Singapore

**Keywords:** thrombosis, tricuspid valve, valve replacement

## Abstract

**Background:**

Prosthetic heart valve implantation over the past 7 decades has revolutionized treatment of valvular heart disease. Mechanical heart valves, although more durable than a bioprosthesis, require lifelong anticoagulation, with risk of valve thrombosis—a serious, life-threatening complication with high morbidity and mortality.

**Case Summary:**

We present a case of prosthetic mechanical tricuspid valve thrombosis that was successfully treated with thrombolysis using tissue plasminogen activator.

**Discussion:**

The incidence of prosthetic mechanical valve thrombosis ranges from 0.1% to 5.7% per year, with higher rates observed in the post-surgical period and for prosthetic tricuspid valves (20%) compared with aortic or mitral valves (0.5%-0.8%). American College of Cardiology /American Heart Association (AHA) 2020 guidelines recommend higher international normalized ratio (INR) for prosthetic tricuspid valve replacement (TVR) and mitral valve replacement 3.0 (INR: 2.5-3.5), as opposed to prosthetic aortic valve replacement 2.5 (INR: 2-3). Current guidelines recommend parenteral anticoagulation, thrombolysis, and redo valve replacement as treatment options for mechanical heart valve thrombosis. Thrombolytic treatment provides a suitable and effective alternative to surgery, with challenges in effective dose administration for right-sided prosthetic valves.

**Take-Home Message:**

An ultraslow thrombolysis regimen can provide a suitable and effective alternative to surgery in treatment of right-sided mechanical valve thrombosis.

## Graphical Abstract

**Figure:**
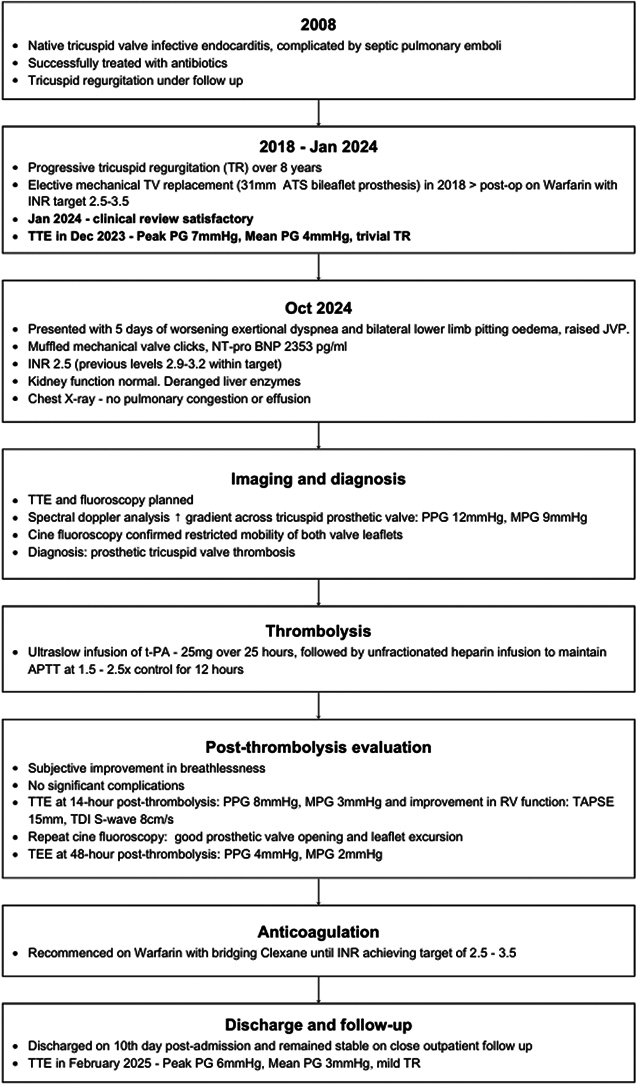

## History of Presentation

A 34-year-old woman with a mechanical TVR presented to the emergency department in October 2024 with worsening exertional dyspnea over 5 days, without chest pain or fever. She reported abdominal discomfort and lower limb swelling and mentioned the mechanical valve clicks were muffled than usual. A few doses of warfarin were missed over the preceding week.

Vital signs were temperature 37.5 °C, pulse rate 98 beats/min, blood pressure 110/78 mm Hg, respiratory rate 19 breaths/min, and oxygen saturation 95% on room air. Cardiac auscultation revealed intermittently audible clicks but no murmurs, jugular venous pressure was 4 cm in semirecumbent position, and respiratory examination was normal. The liver edge was palpable on abdominal examination, with no clinically demonstrable ascites. Bilateral dependent edema was evident up to the mid-calves.

## Past Medical History

**Figure 1:**
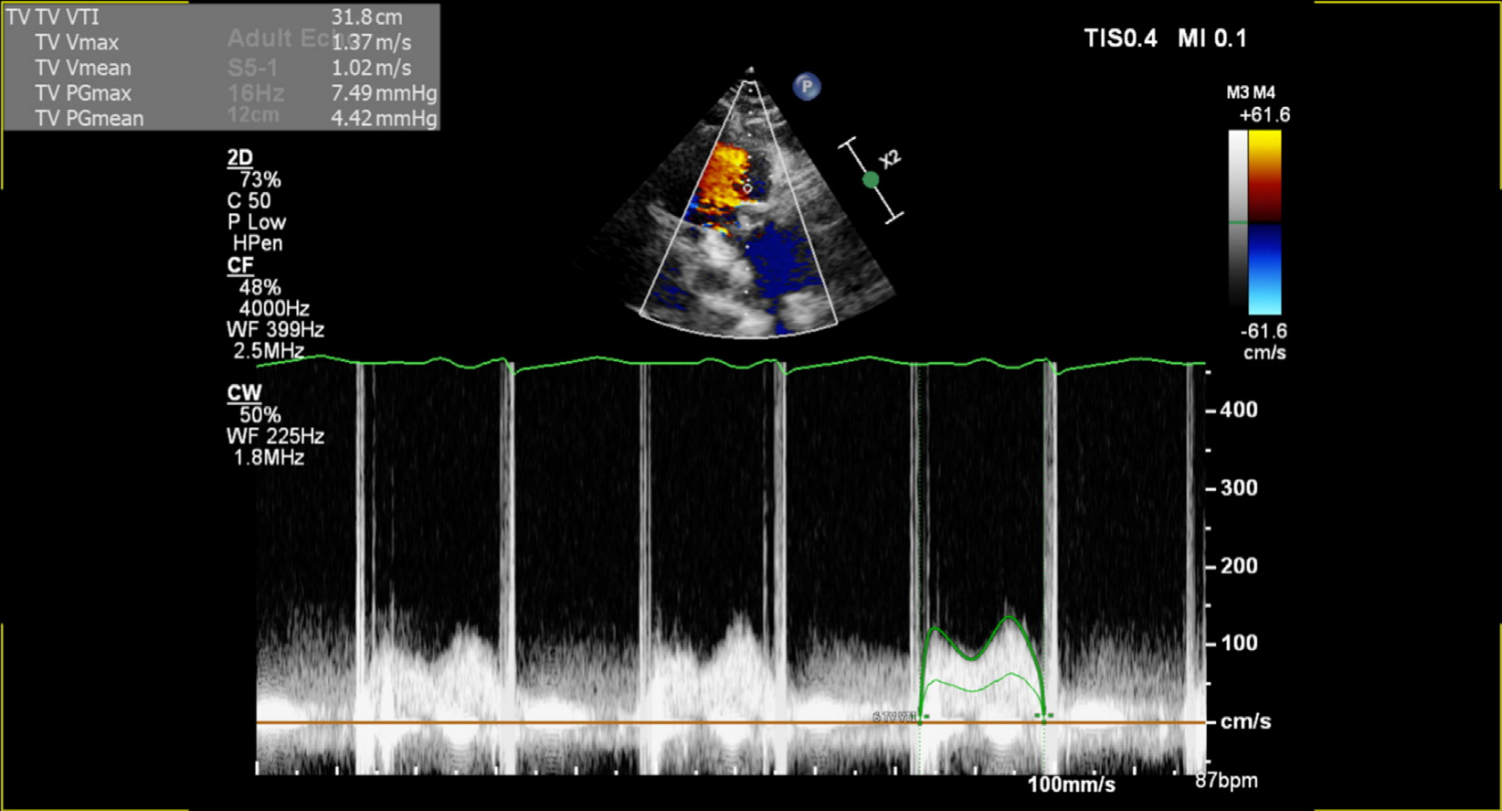
Baseline Transthoracic Echocardiography of Mechanical Tricuspid Valve Replacement With Pulse Wave Doppler in 2023

The patient has asthma that is well controlled on inhalers and has a previous history of intravenous drug abuse. In 2008, at 18 years old, she developed native tricuspid valve endocarditis complicated by septic pulmonary emboli, which were successfully treated with intravenous antibiotics. At regular outpatient follow-up appointments, serial echocardiograms were obtained to monitor the resultant tricuspid valve regurgitation. The valvular regurgitation progressed over the following decade leading to mechanical TVR surgery performed in 2018 at age 28 years implanting a 31-mm ATS bileaflet prosthesis (ATS Medical). Significant tricuspid annular dilatation and leaflet morphology rendered the native valve unsuitable for repair. The patient's young age, limited durability of the bioprosthetic valve, and the need for multiple redo valve procedures over the patient's lifetime were the key contributors to the decision to implant a mechanical prosthesis. She remained under annual follow-up, with anticoagulation using warfarin with target INR of 2.5 to 3.5. Transthoracic echocardiography (TTE) performed in December 2023 confirmed satisfactory gradients: peak pressure gradient 7 mm Hg and mean pressure gradient 4 mm Hg. There was trivial tricuspid regurgitation (Figure 1, Video 1).

## Differential Diagnosis

The differential diagnosis included right-sided congestive cardiac failure, prosthetic valve dysfunction, and endocarditis.

## Investigations

A 12-lead electrocardiogram showed sinus rhythm at 70 beats/min, with known partial right bundle branch block. Cardiac enzymes were unremarkable with INR 2.5 (preceding levels: 2.9-3.2), and renal parameters were normal with creatinine of 76 μmol/L (normal range: 37-75 μmol/L). Liver function tests showed transaminitis with raised serum alanine transaminase 143 U/L (normal range: 6–66 U/L) and aspartate transaminase 68 U/L (normal range: 12-42 U/L). Alkaline phosphatase level was normal at 67 U/L (normal range: 39-99 U/L). N-terminal pro–B-type natriuretic peptide was elevated at 2,353 pg/mL (normal range: <150 pg/mL). Chest radiograph revealed prominent hilar vascular markings, and no interstitial edema or pleural effusion.

**Figure 2:**
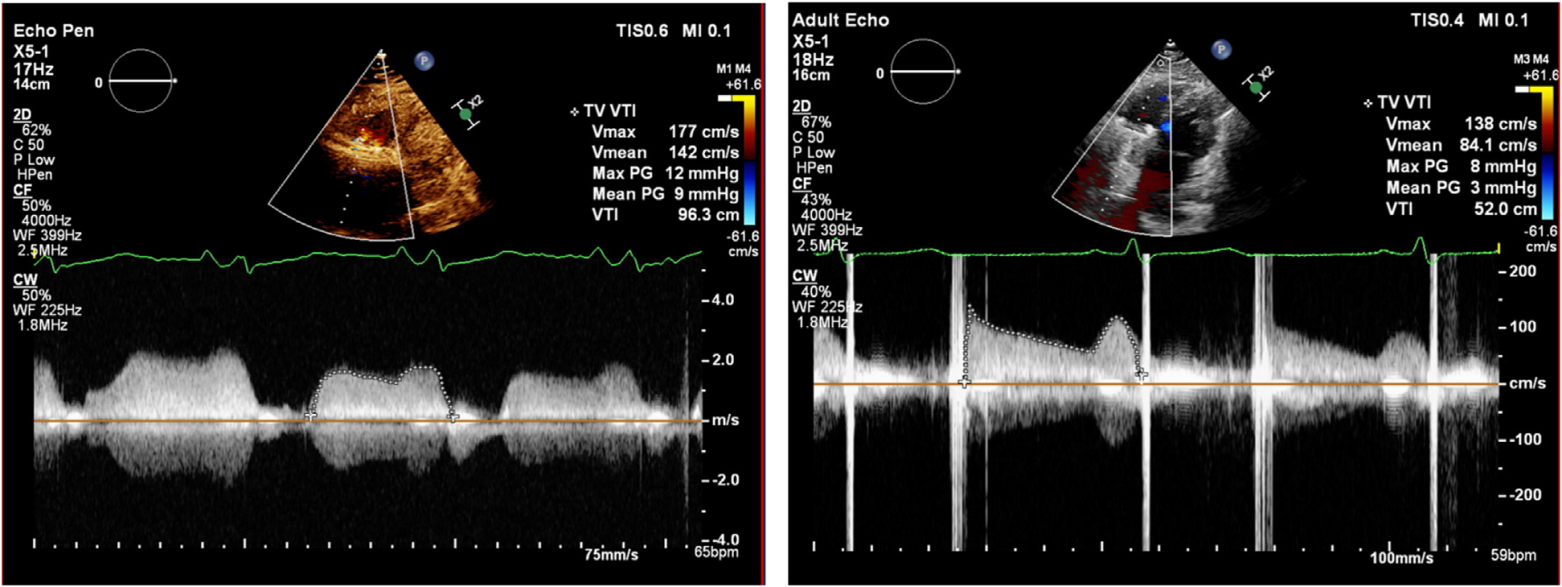
Pulsed Wave Doppler Across Mechanical Tricuspid Valve Replacement Pre-thrombolysis (left) and post-thrombolysis (right).

TTE revealed well-seated tricuspid valve prosthesis, with increased transvalvular gradients, peak pressure gradient 12 mm Hg, and mean pressure gradient 9 mm Hg (Figure 2, Video 2). These were significantly elevated compared with previous TTE performed a year ago (peak pressure gradient 7, mean pressure gradient 4 mm Hg). Mild to moderate tricuspid regurgitation was also noted. Right ventricle function was reduced, tricuspid annular plane systolic excursion was 14 mm, and tissue Doppler imaging S-wave was 8.7 cm/s. Differential diagnoses included mechanical valve obstruction caused by valve thrombosis, pannus, degeneration, and recurrent endocarditis. Pannus was less likely, given the relatively acute onset of symptoms. The absence of pyrexia with normal inflammatory markers made endocarditis an unlikely contributor to the presentation. Valve fluoroscopy subsequently confirmed restricted mobility of both valve leaflets (Video 3), confirming the diagnosis of prosthetic valve thrombosis.

## Management

The patient’s presentation and imaging findings were discussed in at the heart team meeting comprising cardiologists, cardiac surgeons, and pharmacists. Redo valve replacement was deemed relatively high risk (EuroSCORE II 8.1%). Thrombolytic therapy was therefore a suitable alternative, using ultraslow infusion, as opposed to slow infusion, of tissue-type plasminogen activator (t-PA). The patient's preference was also noninvasive treatment strategy, accepting the risks involved, including recurrent prosthetic valve thrombosis. Ultraslow infusion of t-PA (25 mg over 25 hours) was administered in the coronary care unit, followed by unfractionated heparin infusion to maintain activated partial thromboplastin time 1.5 to 2.5 times control for 12 hours. TTE performed 14 hours post-thrombolysis revealed improvement in all measured parameters, peak pressure gradient of 8 mm Hg, and mean pressure gradient of 3 mm Hg (Figure 2, Video 4A). Right ventricular function improved (tricuspid annular plane systolic excursion 15 mm, tissue Doppler imaging S-wave 8 cm/s). Cinefluoroscopy confirmed good prosthetic valve opening and disc motion (Video 5).

**Figure 3:**
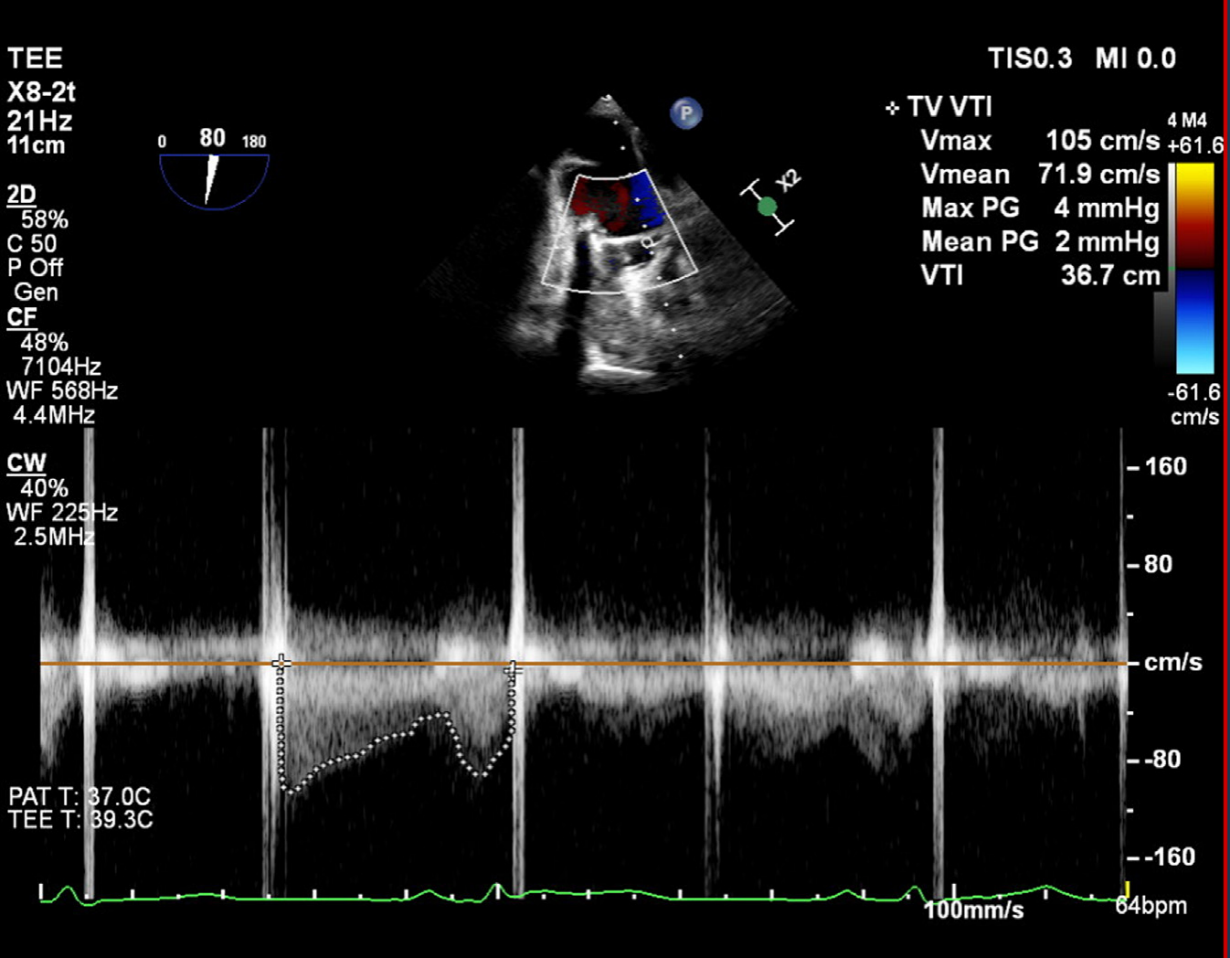
Pulsed Wave Doppler Across Mechanical Tricuspid Valve Replacement on Transesophageal Echocardiography, 48 h Post-Thrombolysis

Transesophageal echocardiography (TEE) performed 48 hours post-thrombolysis confirmed symmetrical movement of both metallic hemidiscs, with no visualized thrombus. Peak pressure gradient further improved to 4 mm Hg, with mean pressure gradient 2 mm Hg (Figure 3, Video 4B, Table 1).

**Table 1:** Summary of Sequential Echocardiographic Imaging and Progress of Prosthetic Tricuspid Valve Gradients

Timing of Echocardiogram	Modality	Peak PG, mm Hg	Mean PG, mm Hg	Tricuspid Regurgitation	TAPSE, mm	TDI RV S-wave, cm/s
On admission	TTE	12	9	Mild to moderate	14	8.7
14 h post-thrombolysis	TTE	8	3	Mild	15	8
48 h post-thrombolysis	TEE	4	2	Trivial		
4 mo post-thrombolysis	TTE	6	3	Mild	19	10

PG = pressure gradient; RV = right ventricle; TAPSE = tricuspid annular plane systolic excursion; TDI = tissue Doppler imaging; TEE = transesophageal echocardiography; TTE = transthoracic echocardiography.

Post-thrombolysis, the patient reported considerable improvement in breathlessness. There were no immediate procedural complications. Anticoagulation with warfarin was restarted with bridging subcutaneous enoxaparin until desirable INR levels (2.5–3.5) were achieved. As subtherapeutic anticoagulation was attributable to medication noncompliance, the therapeutic target was kept unchanged. She was discharged on the 10th day post-admission and has remained stable during close outpatient follow-up.

**Visual Summary:**
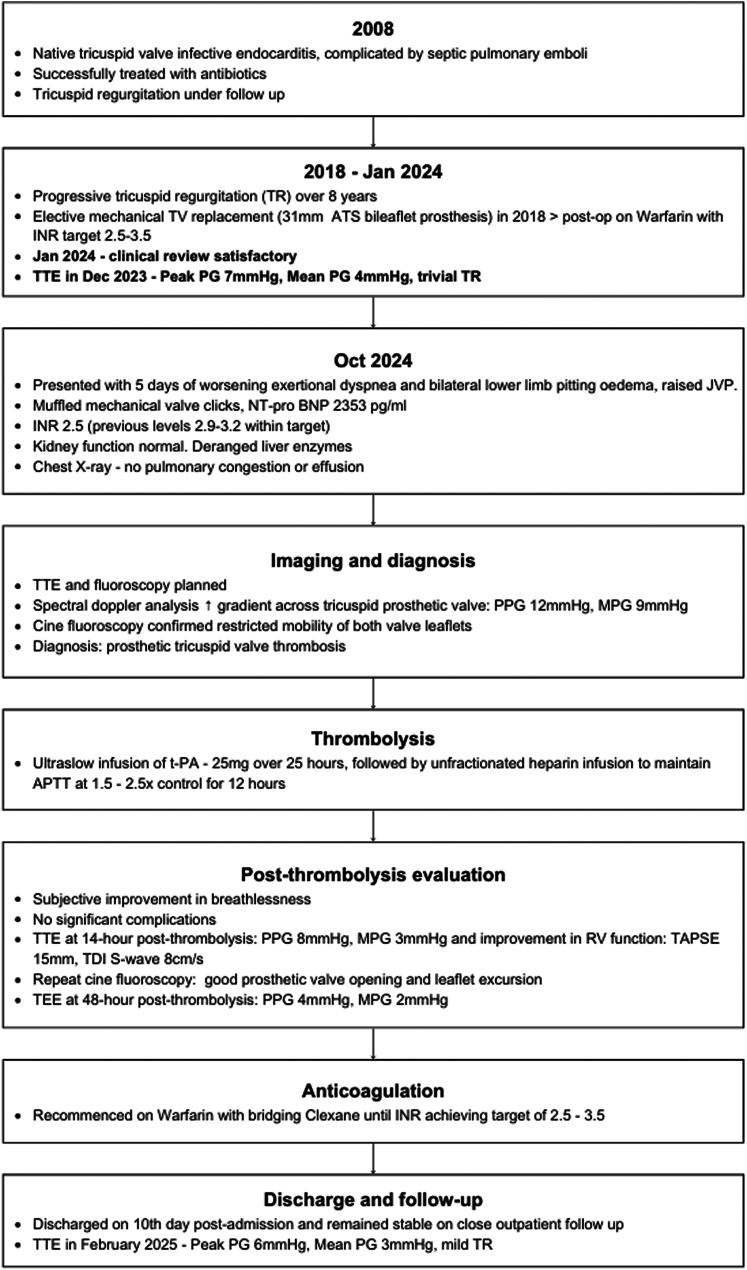
Flowchart Summarizing Events From Admission to Discharge

## Outcome and Follow-Up

The patient has remained clinically well with anticoagulation in therapeutic range. An up-to-date TTE in February 2025 confirmed satisfactory prosthetic valve gradients: peak pressure gradient 6 mm Hg, mean pressure gradient 3 mm Hg, with mild tricuspid valve regurgitation.

## Discussion

Mechanical valve thrombosis is a well-recognized complication in prosthetic tricuspid valves. The presentation ranges from breathlessness, chest discomfort to cardiogenic shock, depending on the acuteness of thrombus formation and resulting valve obstruction, with or without associated regurgitation. A high index of suspicion, detailed history of the onset and progression of symptoms, hemodynamic assessment, and anticoagulation trend all contribute to the clinical diagnosis. Relatively acute onset of symptoms, noncompliance with anticoagulants, and subtherapeutic INR trend are more suggestive of thrombus, whereas pannus overgrowth tends to manifest in a subacute manner. Distinction between thrombosis and pannus is essential, as thrombolytic therapy is indicated in thrombosis, but contraindicated in pannus overgrowth. In addition, low therapeutic index of thrombolysis and risk of major bleeding make the differentiation crucial.

TTE provides an assessment of prosthetic valve structure, disc opening, and mobility (within the limitations of acoustic reverberations), alongside quantification of transvalvular gradients. Elevated peak tricuspid velocity >1.9 m/s and mean pressure gradient >6 mm Hg, regardless of valve appearance are suggestive of obstructive valve thrombosis.^1^ TEE, especially three-dimensional TEE, allows comprehensive analysis of the prosthetic valve with improved visualization of valve position, disc opening, mobility, and paravalvular structures. Spectral Doppler analysis enables quantification of gradients, supporting the data obtained on TTE.

Cine fluoroscopy provides real-time assessment of disc motion, thereby providing additional information, especially if echocardiographic assessment has been inconclusive or suboptimal due to acoustic reverberation.^2^

Echocardiography and fluoroscopy are sufficient in making the diagnosis of prosthetic valve thrombosis, and multimodality imaging is rarely required. In a small number of cases in which the diagnosis is not established, cardiac computed tomography may provide further information including attenuation values. An attenuation value of >145 Hounsfield units is more likely to represent pannus, whereas a value below this represents thrombus.^3^ As our patient had sufficient evidence to support the diagnosis of prosthetic valve thrombosis on TTE and fluoroscopy, computed tomography was not performed.

## Current evidence in management of mechanical valve thrombosis

Current management strategies for mechanical valve thrombosis include parenteral anticoagulation, thrombolysis, or redo valve replacement surgery. With the lack of randomized controlled trials, treatment options of prosthetic valve thrombosis remain unclear.^4^ In addition, the patient's clinical state, comorbidities, risk of surgery and bleeding, thrombus burden, and degree of valve dysfunction (obstruction/regurgitation) are the leading contributors to decision making. The European Society of Cardiology and AHA have conflicting recommendations with regard to the use of thrombolytics, which adds to the challenge of therapeutic decision making. The 2020 AHA guidelines support the use of low-dose thrombolysis for left-sided mechanical valve thrombosis but do not elaborate on its efficacy in right-sided (ie, tricuspid) prosthetic valve thrombosis.^4^ In contrast, the European Society of Cardiology and European Association of Cardiothoracic Surgery prioritize surgical intervention for right-side prosthetic valve thrombosis. Due to the paucity of available data, a multidisciplinary heart team approach is essential in deciding the optimal treatment option, tailored to the individual patient’s presentation and imaging findings.

Several trials have been performed exploring the role of thrombolysis in treatment of obstructive prosthetic valve thrombosis (Supplemental Table 1). In the recent multicenter, prospective, observational HATTUSHA study, thrombolytic therapy was performed in 83 (52.5%) patients versus surgery in 75 (47.5%) patients, of which 13 patients had tricuspid valve thrombosis; 9 (11%) had thrombolysis and 4 (5.5%) underwent surgery.^5^ Thrombolysis was performed using slow (6 hours) vs ultraslow (25 hours) infusion of low-dose t-PA without initial loading dose, in repeated doses, followed by unfractionated heparin infusion post-thrombolysis to achieve activated partial thromboplastin time 1.5 to 2.5 times the control. Success in patients with obstructive prosthetic valve thrombosis undergoing thrombolysis was defined as improved Doppler parameters of valve hemodynamics, reduction in thrombus dimensions (diameter/area) by 75%, and improvement in symptoms. Complete success required all 3 criteria, whereas partial success comprised 2 of 3 criteria being met. The reduced complication rates of thrombolytic treatment versus surgery (total complications: 12% vs 62%; major bleeding: 2.4% vs 9.3%; 3-month mortality: 2.4% vs 18.7%) make thrombolysis an appealing and viable treatment option in patients with obstructive tricuspid prosthetic valve thrombosis.^5^ Despite the small sample size (13 of 158), this observational study provides the best available evidence to support thrombolytic therapy as a suitable/effective treatment option in mechanical tricuspid valve thrombosis.

## Conclusions

Despite the frequency of valvular disease, TVR in isolation is not a commonly performed procedure, with controversies surrounding the choice between a biological or mechanical prosthesis. Bioprosthetic valves are preferred for right-sided valve replacement with the advantages of no anticoagulation required and probable suitability for percutaneous valve intervention subsequently in the event of prosthesis dysfunction. However, the failure rates owing to degradation lead to a second replacement. In comparison, mechanical tricuspid prostheses are more durable and have low reoperation rates, but they require lifelong anticoagulation and carry the risk of valve thrombosis.

Obstructive prosthetic valve thrombosis is a well-established complication of mechanical valves, with manifestations ranging from heart failure to cardiogenic shock. A high index of suspicion and early diagnosis are crucial in effective management. Thrombolysis has proven to be a safe treatment strategy in obstructive left-sided mechanical valve thrombosis in various trials, but there is a paucity of data in right-sided mechanical valve thrombosis (Supplemental Table 1). In spite of the challenges of conducting a randomized controlled trial, observational studies and case reports have described successful outcomes using various thrombolytics agents (Supplemental Table 2). The HATTUSHA observational study, comparing the use of slow (6 hours) and ultraslow (25 hours) infusion of alteplase infusion in 158 patients with obstructive prosthetic valve thrombosis, including 9 cases of prosthetic tricuspid valve, showed high success rates and fewer complications, including major bleeding and mortality, at 3 months.^5^ Despite the small number of cases with tricuspid valve thrombosis (13 of 158) in this nonrandomized observational study, the findings support the use of thrombolytic therapy as a suitable and effective alternative to surgery in cases of prosthetic tricuspid valve thrombosis. Nonetheless, further large-scale randomized controlled trials are required for validation of these findings.

**Take-Home Message**

• Our case supports the benefits of thrombolytic therapy using ultraslow recombinant tPA infusion protocol (25 mg over 25 hours, followed by unfractionated heparin infusion for 12 hours) in obstructive mechanical tricuspid valve thrombosis.

## Funding Support and Author Disclosures

This work was not supported by any grants, contracts, or other forms of financial support. The author(s) have no relationship with industry to disclose.

## Appendix

Video 1 Baseline Transthoracic Echocardiography of Mechanical Tricuspid Valve Replacement With Pulsed Wave Doppler in 2023

Video 2 Transthoracic Echocardiography and Pulsed Wave Doppler of Mechanical Tricuspid Valve Replacement Pre-thrombolysis

Video 3 Fluoroscopy of Mechanical Tricuspid Valve Replacement Pre-thrombolysis

Video 4A Transthoracic Echocardiography and Pulsed Wave Doppler of Mechanical Tricuspid Valve Replacement Post-thrombolysis

Video 4B Three-Dimensional Transesophageal Echocardiography of Mechanical Tricuspid Valve Replacement 48 Hours Post-thrombolysis

Video 5 Fluoroscopy of Mechanical Tricuspid Valve Replacement Post-thrombolysis
