# Supplementary material for: Ultraslow Thrombolysis of Mechanical Tricuspid Prosthetic Thrombosis
Source: JACC Case Rep. 2025 Jul 16;30(19):104211. doi: 10.1016/j.jaccas.2025.104211 (PMC12441459; doi:10.1016/j.jaccas.2025.104211)
Supplement: Supplementary Table 1-2 [file mmc7.docx]

**Supplemental Table 1: Trials/ Studies related to thrombolytic therapy in prosthetic heart valve thrombosis.**

| **First Author** | **Year** | **Objective** | **Finding/Conclusion** |
| --- | --- | --- | --- |
| Özkan et al.^1^ | 2022 | **The Multicentre HATTUSHA Study**  Compared the effectiveness and safety of thrombolysis and surgery in treating patients with obstructive mechanical prosthetic valve thrombosis (PVT) | - Patients treated with thrombolysis (52.5% of participants) received low-dose tissue plasminogen activator (tPA), infused slowly over 6–25 hours, with a total dose reaching 59 mg (median). - The thrombolysis success rate was 90.4%, with a 3-month mortality rate of 2.4%, compared to 18.7% in the surgery group. - Thrombolysis resulted in fewer major complications (2.4%) compared to surgery (9.3%). |
| Özkan et al.^2^ | 2015 | **The Ultra-slow PROMETEE trial**  Evaluated the use of ultra-slow, low-dose thrombolytic therapy (25mg of alteplase over 25-hours) for treating prosthetic mechanical valve thrombosis in 114 patients. | - Overall success rate of thrombolysis was 90%, - Low complication rate of 6.7% (including 3.3% major nonfatal and 0.8% mortality). - Predictors for failure included higher NYHA class and larger thrombus area, while complications were more likely in patients with atrial fibrillation​ |
| Castilho et al.^3^ | 2014 | **Thrombolytic Therapy or Surgery for Valve Prosthesis Thrombosis: Systematic Review and Meta-Analysis**  Systematically compared thrombolysis to surgery in managing prosthetic valve thrombosis, analysing from 48 observational studies, n=2302. | - Thrombolysis had lower mortality (6.6%) compared to surgery (18.1%), though with a higher risk of embolic events (12.8% vs. 4.6% for surgery). Both treatments had similar success rates (~80%). The findings suggest thrombolysis could be a viable option in high-risk surgical candidates, though further trials are needed to confirm these results conclusively. |
| Özkan et al.^4^ | 2013 | **The TROIA Trial**  Evaluated different thrombolytic regimens for prosthetic valve thrombosis (PVT)  Group I: High-dose streptokinase with bolus.  Group II: Low-dose streptokinase, continuous infusion.  Group III: Bolus-free low-dose alteplase infusion.  Group IV: Ultraslow infusion of low dose alteplase.  Group V: Slow infusion of 25 mg alteplase repeated if necessary. | - Slow & low dose Alteplase infusion regimen showed highest overall success rate (83.2%), fewest complications (10.5%), no mortality. |
| Tong et al.^5^ | 2004 | **The International PRO-TEE Registry**  Evaluated the role of TEE in risk stratification for thrombolysis in prosthetic valve thrombosis (PVT) | - Thrombus size > 0.8cm^2^ & a prior history of stroke were identified as independent predictors of complications. - Thrombus area > 0.8cm^2^ was associated with higher risk of complications, irrespective of NYHA class. - Each 1cm^2^ increase in thrombus size correlated with 2.4-fold increase in risk of complications. - Study suggested patients with smaller thrombi (=/<0.8cm^2^) and lower NYHA class may be ideal candidates for thrombolysis as 1^st^-line therapy |

**Supplemental Table 2: Summary of Case Studies of Mechanical TV Thrombosis treated with tPA, success rates and complications**^a^

| **First author** | **Year** | **N** | **Doses/Regime** | **Success rate** | **Safety/ Complication** |
| --- | --- | --- | --- | --- | --- |
| Haraf et al.^6^ | 2024 | 1 | - IV Alteplase 25mg infusion over 25 hours, repeated 1 time. | 100% | - Not reported |
| Ozkan et al.^7^ | 2022 | 9 | - IV Alteplase 25mg infusion over 6 hours. repeat up to 6 times as needed. Max 150mg - IV Alteplase 25mg infusion over 25 hours, repeat up to 8 times as needed. Max 200mg | 90.4% (overall - all valves type) | - Overall (all valves) minor complications: thrombolysis (8.4%) vs surgery (38.7%) - Overall (all valves) major complications: thrombolysis (6%) vs surgical (41.3%). - 3-month mortality: thrombolysis (2.4%) vs surgical (18.7%). |
| Zaghloul et al.^8^ | 2018 | 1 | - IV Alteplase bolus of 2mg followed by continuous infusion at 1mg/hour for up to 24-hour | Treatment failure. | - Not specified |
| Ozkan et al.^2^ | 2015 | 4 | - IV Alteplase 25mg over 25-hour infusion, repeat up to 8 times as needed. Max 200mg. | 100% | - Overall (all valves) complication rate 6.7% - 3.3% non- fatal major complications - 2.5% minor complications - 0.8% mortality |
| Taherkhani et al.^9^ | 2015 | 8 | Alteplase or Streptokinase   - <18 y/o - IV Alteplase - <30kg - IV Alteplase given without loading dose. - >30kg - IV Alteplase - 15mg loading dose over 10mins, followed by 35mg over 30mins and 50mg over 1hour. - IV Streptokinase - 200,000U bolus over 30 minutes followed by 100,000U/hr infusion for at least 6 hours, up to 72 hours | 75% | - Fever (12.5%) - Nausea & vomiting (6.3%) - Minor gum bleeding (6.3%) - 1 in-hospital-death unrelated to thrombolytic complications. |
| Ozkan et al.^4^ | 2013 | 14 | Alteplase and Streptokinase regimes   - Group I: High-dose IV streptokinase bolus. - Group II: Low dose IV streptokinase infusion. - Group III: Bolus-free low-dose IV Alteplase infusion. - Group IV: Ultraslow infusion of low-dose IV Alteplase. - Group V: Slow infusion of 25 mg IV Alteplase without a bolus, repeated if necessary. | 78.6% | Overall (all valves) complication rate (18.6%)   - Group I - 37.5%, - Group II - 24.4% - Group III - 33.3% - Group IV - 29.6% - Group V - 10.5% |
| Yaminisharif et al.^10^ | 2012 | 3 | Case 1 (3 times recurrent thrombosis)   - IV Streptokinase 250,000 U loading dose, followed by 100,000 U/h over 48 hours. - IV Tenecteplase initial 15 mg bolus, followed by 20 mg for 4 hours. - IV Tenecteplase 35mg bolus   Case 2   - IV Steptokinase 250,000 U loading dose, followed by 100,000 U/h for 48 hours.   Case 3   - N/A - clot surgically removed | #1 - 100%  #2 - Failed  #3 - N/A | - #1 - not specified - #2 - not specified - #3 - N/A |
| Zhang et al.^11^ | 2012 | 1 | - Day 1 - Catheter-directed Alteplase 2mg bolus, followed by 6mg over 6 hours. - Day 2 - Catheter-directed Alteplase infusion of 8mg over 6 hours | 100% | - Low grade fever and fatigue |
| Kaul et al.^12^ | 2009 | 1 | - IV Alteplase infusion - 100mg infusion over 3 hours | 100% | - No reported |
| Roudaut et al.^13^ | 2009 | 2 | - Not specified | 100% | - No reported |
| Caceres-Loriga et al.^14^ | 2006 | 9 | - IV Streptokinase 250,000U over 30mins, followed by infusion of 100,000 IU/hr. Max 72 hours | 100% | - Overall (all valves) complication rate 22.1% |
| Tong et al.^5^ | 2004 | 15 | - IV Streptokinase infusion over 12 to 48 h, with occasional extension to 72 to 120-hours (dose not specified) - IV Urokinase - slow infusion over 6 to 48 hours (dose not specified) - IV Alteplase - 10mg bolus followed by 90mg/2-6hours | 93.3% | Overall (all valves),   - major bleeding in 2 patients - embolic events in 5 patients - 4 patients died, - 11 pts re-thrombosis - 10 retreated successfully with thrombolysis |
| Roudaut et al.^15^ | 2003 | 2 | - Not specified | 100% | Overall (all valves) complication rate 25.2%   - Major bleeding 4.7% - 2 brain hemorrhage (1 death) - 4 peripheral hemorrhage (2 deaths) - Systemic embolism 15% - TIA (n=6), stroke (n=8), peripheral embolism (n=5) - Death 11.8% |
| Ramos et al.^16^ | 2003 | 4 | - IV Streptokinase bolus of 250,000U followed by 100.000U/hour | 75% | Overall (all valves)   - Fatal bleeding (5.8%) - Embolic event (17.6%) - Re-thrombosis index 33% in a mean f/up of 42 months |
| López et al.^17^ | 2002 | 2 | - IV Streptokinase bolus of 250,000U followed by 100,000U/hour | 93.3% (across all valves) | Overall (all valves)   - No major bleeding events. - 2 peripheral embolisms - 1 minor peripheral bleeding |

**^a^** References are listed in the Supplemental Appendix. Only cases published since year 2000 are included.

**Bibliography**

1. Özkan, M. *et al.* Thrombolysis or Surgery in Patients With Obstructive Mechanical Valve Thrombosis: The Multicenter HATTUSHA Study. *J. Am. Coll. Cardiol.* **79**, 977–989 (2022).

2. Özkan, M. *et al.* Ultraslow thrombolytic therapy: A novel strategy in the management of PROsthetic MEchanical valve Thrombosis and the prEdictors of outcomE: The Ultra-slow PROMETEE trial. *Am. Heart J.* **170**, 409–418 (2015).

3. Castilho, F. M., De Sousa, M. R., Mendonça, A. L. P., Ribeiro, A. L. P. & Cáceres‐Lóriga, F. M. Thrombolytic therapy or surgery for valve prosthesis thrombosis: systematic review and meta‐analysis. *J. Thromb. Haemost.* **12**, 1218–1228 (2014).

4. Özkan, M. *et al.* Comparison of different TEE-guided thrombolytic regimens for prosthetic valve thrombosis: the TROIA trial. *JACC Cardiovasc. Imaging* **6**, 206–216 (2013).

5. Tong, A. T. *et al.* Transesophageal echocardiography improves risk assessment of thrombolysis of prosthetic valve thrombosis: results of the international PRO-TEE registry. *J. Am. Coll. Cardiol.* **43**, 77–84 (2004).

6. Haraf, R., Atkinson, T., Elizabeth Le, D.-T. & Arora, B. Mechanical Tricuspid Valve Thrombosis Successfully Treated With Tissue Plasminogen Activator. *JACC Case Rep.* **29**, 102180 (2024).

7. Özkan, M. *et al.* Thrombolysis or Surgery in Patients With Obstructive Mechanical Valve Thrombosis. *J. Am. Coll. Cardiol.* **79**, 977–989 (2022).

8. Zaghloul, A., Iorgoveanu, C., Desai, A., Silkowski, M. & Balakumaran, K. Prosthetic Tricuspid Valve Thrombosis. *Cureus* **10**, e2928 (2018).

9. Taherkhani, M. *et al.* Thrombolytic Therapy for Right-Sided Mechanical Pulmonic and Tricuspid Valves: The Largest Survival Analysis to Date. *Tex. Heart Inst. J.* **42**, 543–547 (2015).

10. Yaminisharif, A., Alemzadeh-Ansari, M. J. & Ahmadi, S. H. Prosthetic tricuspid valve thrombosis: three case reports and literature review. *J. Tehran Heart Cent.* **7**, 147–155 (2012).

11. Zhang, D. Y. *et al.* Case study and review: treatment of tricuspid prosthetic valve thrombosis. *Int. J. Cardiol.* **162**, 14–19 (2012).

12. Kaul, P., Adluri, K., Javangula, K. & Baig, W. Successful management of multiple permanent pacemaker complications--infection, 13 year old silent lead perforation and exteriorisation following failed percutaneous extraction, superior vena cava obstruction, tricuspid valve endocarditis, pulmonary embolism and prosthetic tricuspid valve thrombosis. *J. Cardiothorac. Surg.* **4**, 12 (2009).

13. Roudaut, R. *et al.* Management of prosthetic heart valve obstruction: fibrinolysis versus surgery. Early results and long-term follow-up in a single-centre study of 263 cases. *Arch. Cardiovasc. Dis.* **102**, 269–277 (2009).

14. Cáceres-Lóriga, F. M. *et al.* Thrombolysis as first choice therapy in prosthetic heart valve thrombosis. A study of 68 patients. *J. Thromb. Thrombolysis* **21**, 185–190 (2006).

15. Roudaut, R. *et al.* Fibrinolysis of mechanical prosthetic valve thrombosis: a single-center study of 127 cases. *J. Am. Coll. Cardiol.* **41**, 653–658 (2003).

16. Ramos, A. I. O. *et al.* Fibrinolytic therapy for thrombosis in cardiac valvular prosthesis short and long term results. *Arq. Bras. Cardiol.* **81**, 393–398, 387–392 (2003).

17. López, H. P. *et al.* Thrombolytic therapy with recombinant streptokinase for prosthetic valve thrombosis. *J. Card. Surg.* **17**, 387–393 (2002).
